# Intermittent Hypoxia-Induced Enhancements in Corticospinal Excitability Predict Gains in Motor Learning and Metabolic Efficiency

**DOI:** 10.21203/rs.3.rs-4259378/v1

**Published:** 2024-04-24

**Authors:** Alysha T. Bogard, Thomas G. Hembree, Aviva K. Pollet, Andrew C. Smith, Stephanie C. Ryder, George Marzloff, Andrew Q. Tan

**Affiliations:** 1 Sensorimotor Recovery and Neuroplasticity Lab at the University of Colorado, Boulder, Dept. of Integrative Physiology, 80309, USA; 2 University of Colorado School of Medicine, Dept. of Physical Medicine and Rehabilitation, Aurora, 80045, USA; 3 Rocky Mountain Regional VA Medical Center, Aurora, 80045, USA; 4 Center for Neuroscience, University of Colorado, Boulder, 80309, USA

## Abstract

Acute intermittent hypoxia (AIH) enhances human motor function after incomplete spinal cord injury. Although the underlying mechanisms in humans are unknown, emerging evidence indicates that AIH facilitates corticospinal excitability to the upper limb. However, the functional relevance of this plasticity remains unexplored, and it is unclear whether similar plasticity can be induced for lower limb motor areas. We recently demonstrated that AIH improves motor learning and metabolic efficiency during split-belt walking. Thus, we hypothesized that AIH increases lower limb excitability and that these enhancements would predict the magnitude of motor learning and the corresponding reductions in net metabolic power. We assessed tibialis anterior (TA) excitability using transcranial magnetic stimulation and quantified changes in spatiotemporal asymmetries and net metabolic power in response to split-belt speed perturbations. We show that AIH enhances TA excitability, and that the magnitude of this facilitation positively correlates with greater spatiotemporal adaptation. Notably, we demonstrate a novel association between increased excitability and reduced net metabolic power during motor learning and savings. Together, our results suggest that AIH-induced gains in excitability predict both the magnitude of motor learning and the associated metabolic efficiency. Determining indices of AIH-induced improvements in motor performance is critical for optimizing its therapeutic reach.

## Introduction

Acute intermittent hypoxia (AIH), involving brief alternating exposures to low-oxygen air, shows promise as an adjuvant to conventional locomotor training to enhance motor function following a motor incomplete spinal cord injury (iSCI)^1–3^. Despite considerable research in rodents^4,5^, the neural mechanisms underlying gains in motor performance remain unclear in humans. Both animal^6^ and human studies^7^ suggest that AIH induces neuroplastic changes that prime the nervous system for further improvements in motor function with neurorehabilitation training. Understanding the neurophysiological underpinnings of AIH-induced plasticity and its functional significance is critical to optimize its therapeutic reach.

One potential mechanism underlying AIH-induced gains in motor performance is enhanced excitability of residual descending motor pathways. Motor-evoked potentials (MEPs) elicited by transcranial magnetic stimulation (TMS) reflect the net excitability of intracortical and transcortical networks synapsing onto corticospinal neurons^8^. Previous studies in able-bodied individuals have shown that AIH may facilitate corticospinal excitability (CSE) to the upper limb^9,10^. However, contrasting evidence indicates that prolonged hypoxic exposures do not alter CSE^11^, and even milder exposures result in inconsistencies in both the time course and stimulus intensity required for such augmentation^9,10^. These discrepancies underscore a critical gap in our understanding of AIH-induced plasticity, including uncertainties regarding how AIH may differentially affect lower limb motor areas that support locomotor control. Furthermore, it remains unclear how increased CSE influences motor performance.

Our recent study demonstrated that AIH enhances spatiotemporal adaptation during a novel split-belt walking task, suggesting that AIH may mediate motor performance through processes involved in motor learning^12^. Such enhancements may be attributed to brain-derived neurotrophic factor (BDNF)-dependent mechanisms known to enhance both CSE and motor learning^13,14^. Evidence from rodent models indicates that moderate AIH exposures increase motoneuron excitability through BDNF-dependent mechanisms^4,5^. Therefore, AIH-induced enhancements in CSE may not only reflect the net changes in excitability but also subserve gains in motor learning.

Converging evidence from human studies reports positive correlations between elevated CSE and enhanced motor learning^15,16^. Gains in CSE may relate to the challenge of the motor learning process itself^17^ rather than specific movement parameters^18^. Notably, tibialis anterior (TA) excitability increases after novel skill training but not after repetitive movement training^19^. Moreover, TA excitability is amplified during late stance of a precision target-matching walking task relative to regular walking^20^. Conversely, disrupting motor cortical processing through repetitive stimulation subsequently lowers CSE and impedes motor retention^21^. Together, this evidence suggests that elevated CSE may serve as a marker of neuroplastic processes that promote motor learning.

Accordingly, this study investigated the effects of five consecutive days of AIH on TA excitability and motor learning. AIH involved 90-second intervals of breathing 9% ± 2% O_2_ alternated with 60-second intervals of 21% ± 2% O_2_ for 15 cycles^3^. We measured changes in resting TA excitability pre-AIH and post-AIH using single-pulse TMS. To assess whether AIH-induced enhancements in CSE influence motor learning without any prior training, participants performed a split-belt walking task after the final AIH dose to eliminate the confound of task familiarity^12^.

We hypothesized that AIH would increase TA excitability and that the magnitude of change would predict the extent of spatiotemporal adaptation during split-belt walking. Since greater spatiotemporal adaptation is associated with decreased metabolic power^22^, with more pronounced reductions after AIH^12^, we further predicted a positive correlation between increased TA excitability and reduced net metabolic power. Bridging the gap between AIH-induced neuroplasticity and its functional significance is critical for maximizing the neurorehabilitative benefits of AIH.

## Results

### AIH Increases Lower Limb Excitability

**Figure 1.**
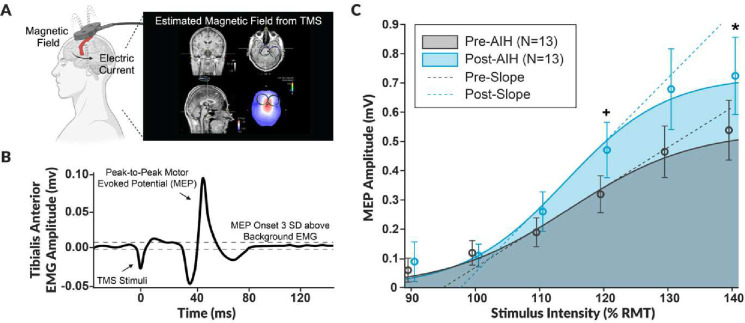
The average transcranial magnetic stimulation recruitment curve pre- and post-acute intermittent hypoxia exposure. **A.** We used single-pulse transcranial magnetic stimulation (TMS) to assess changes in corticospinal excitability to the tibialis anterior before and after five consecutive days of acute intermittent hypoxia (AIH). We applied TMS over the scalp to target the lower limb motor area of the primary motor cortex. We utilized a neuronavigational system to ensure trial-to-trial consistency in the stimulation vector. The hotspot corresponded to the location that elicited the lowest threshold and shortest latency motor evoked potential (MEP) amplitude. **B.** The MEP was recorded using surface electromyography (EMG) placed over the tibialis anterior muscle belly. We utilized the peak-to-peak MEP amplitude as an index to evaluate changes in corticospinal excitability, where the onset was identified as the first frame to exceed three standard deviations of the pre-stimulus background EMG. **C.** The average recruitment curve for thirteen participants was generated by plotting the peak-to-peak MEP amplitude versus the normalized stimulus intensity based on 90% to 140% of the participants’ resting motor threshold (RMT) pre-AIH (grey) and post-AIH (blue). The dashed line represents the Boltzmann slope, the shaded area depicts the area under the curve, and the bars represent standard error. The significance levels of an ANOVA comparing pre-AIH and post-AIH at the stimulus intensities of 100% RMT, 120% RMT, and 140% RMT are denoted as * p < 0.05 and + p = 0.05.

Thirteen able-bodied subjects participated in a within-subjects design to assess changes in CSE after 5 consecutive days of AIH. We generated recruitment curves by plotting the peak-to-peak MEP amplitudes against the normalized stimulus intensities based on the participant’s resting motor threshold (RMT) (Fig. 1)^11^. To assess changes in CSE, we quantified TMS indices such as changes in the maximum MEP amplitude (MEP_max_), the area under the recruitment curve, the peak slope of the recruitment curve, and RMT.

**Figure 2.**
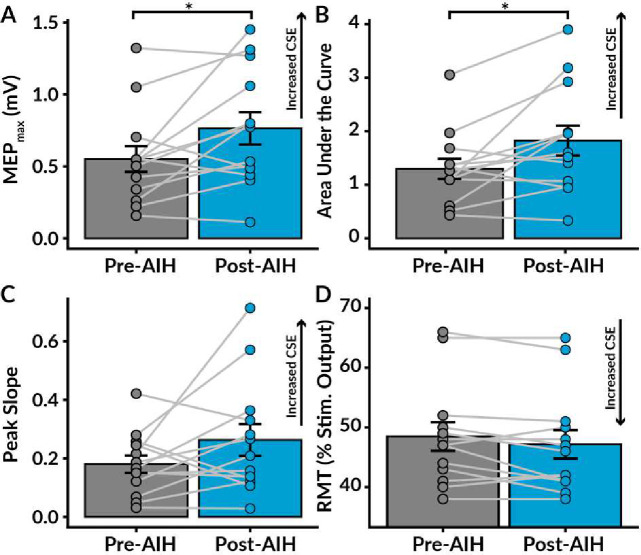
Average corticospinal excitability pre- and post-acute intermittent hypoxia exposure. We evaluated changes in corticospinal excitability (CSE) pre- to post-acute intermittent hypoxia (AIH) by measuring alterations in the maximum motor evoked potential amplitude (MEP_max_), area under the recruitment curve, peak slope of the recruitment curve, and resting motor threshold (RMT; as a percent of the maximal stimulator output). **A.** The average MEP_max_ significantly increased following AIH, indicating increased CSE. **B.** The area under the recruitment curve, an index of net excitability from 90% to 140% of RMT, also significantly increased post-AIH. **C.** There was no significant change in peak slope after AIH. **D.** The RMT decreased after AIH, suggesting increased CSE. However, this change only approached significance (p = 0.088). The bars represent standard error. Significance levels are denoted as *** p < 0.001, ** p < 0.01, and * p < 0.05.

We observed a significant increase in TA excitability after AIH (Fig. 2), as shown by significant enhancements in both MEP_max_ (W = 14, p = 0.027) and the area under the recruitment curve (W = 13, p = 0.021). Additionally, we observed a decrease in RMT after AIH, indicating a lower threshold to excite the TA; however, this change only approached significance (W = 44.5, p = 0.088). In contrast, peak slope (W = 25, p = 0.168) and maximal voluntary contraction (t(12) = -0.78, p = 0.450) did not change after AIH.

Furthermore, a repeated measures ANOVA comparing the MEP amplitude pre-AIH and post-AIH at the stimulus intensities of 100% RMT, 120% RMT, and 140% RMT, showed a significant main effect of both timepoint (F(1, 12) = 6.66, p = 0.024) and stimulus intensity (F(1.18, 14.19) = 36.29, p < 0.001) on MEP amplitude, as well as a significant interaction between timepoint and stimulus intensity (F(1.49, 17.86) = 4.79, p = 0.030). Tukey’s post-hoc comparisons indicated a significant increase in MEP amplitude from pre- to post-AIH for the stimulus intensities of 120% RMT (t(12) = −2.18, p = 0.050) and 140% RMT (t(12) = −2.75, p = 0.018). These findings collectively demonstrate that five consecutive days of AIH significantly enhanced TA excitability.

### Background EMG

To ensure stable levels of pre-stimulus muscle activation, we compared the mean background EMG recorded 200 ms preceding the TMS stimuli^23^. We did not observe any statistically significant differences in background EMG activity between pre-TMS and post-TMS sessions (t(12) = −0.72, p = 0.486). This observation indicates that the variability in background EMG is not a significant factor contributing to any observed changes in the TA MEP amplitude pre- to post-AIH.

### Motor Learning: Spatiotemporal Asymmetry

**Figure 3.**
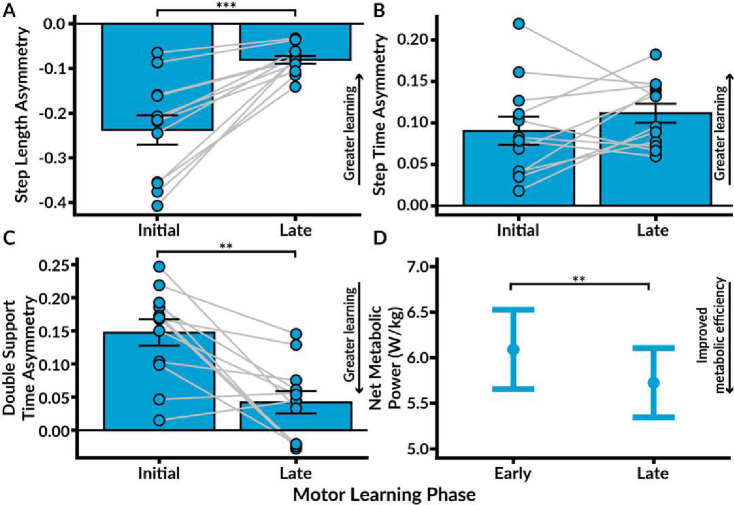
Motor Learning: Spatiotemporal and metabolic adaptation during the first exposure to split-belt walking. We quantified motor learning by measuring the extent of spatiotemporal adaptation from the first five strides (‘initial’) to the final twenty strides (‘late’) of the first exposure to split-belt walking (‘Adapt 1’). Additionally, we assessed the magnitude of metabolic adaptation by comparing the second minute (‘early’) of Adapt 1 to the last minute (‘late’). **A.** We observed substantial reductions in step length asymmetry during Adapt 1, indicating spatial motor learning. **B.** In contrast, step time asymmetry remained high without significant changes. **C.** Double support time asymmetry significantly decreased from initial Adapt 1 to late Adapt 1, consistent with temporal motor learning. **D.** Net metabolic power significantly reduced during Adapt 1, suggesting improved metabolic efficiency. The circles and light grey lines depict individual data. The bars represent standard error. Significance levels are denoted as *** p < 0.001, ** p < 0.01, and * p < 0.05.

We assessed motor learning by quantifying the extent of spatiotemporal adaptation from the first five strides (‘initial learning’) to the last twenty strides (‘late learning’) of two, 300-stride split-belt walking conditions, Adapt 1 and Adapt 2 (Fig. 3A–C). For both adaptation conditions, the perturbation was set at a 2:1 belt speed ratio. The spatiotemporal parameters of motor learning we analyzed included step length asymmetry (SLA), step time asymmetry (STA), and double support time asymmetry (DSA). Values that converged with the adaptation patterns extensively observed in able-bodied individuals reflected greater ‘learning.’ Specifically, we characterized motor learning by reductions in SLA and DSA towards symmetry values of 0, coupled with increases in STA towards positive asymmetry^24,25^. One participant was excluded from all gait analyses due to utilizing a running strategy with a distinct aerial phase.

We found that participants adapted their interlimb coordination during Adapt 1 and Adapt 2 to minimize SLA and DSA, while maintaining an increased STA. A two-way ANOVA revealed significant main effects of both condition (F(1, 11) = 22.99, p < 0.001) and learning phase (F(1, 11) = 36.15, p < 0.001) on SLA, with a significant interaction between condition and learning phase (F(1,11) = 15.43, p = 0.002). Tukey’s post-hoc comparisons demonstrated a significant decrease in SLA from initial Adapt 1 to late Adapt 1 (t(11) = −5.65, p < 0.001) and from initial Adapt 2 to late Adapt 2 (t(11) = −4.73, p < 0.001). Conversely, for STA, a two-way ANOVA only observed a significant main effect of condition (F(1, 11) = 7.78, p = 0.018). However, Tukey’s post-hoc comparisons did not indicate any significant changes in STA between initial Adapt 1 to late Adapt 1 (p = 0.214), nor between initial Adapt 2 to late Adapt 2 (p = 0.784). For DSA, a two-way ANOVA showed significant main effects of condition (F(1, 11) = 38.79, p < 0.001) and learning phase (F(1, 11) = 14.89, p = 0.003), with a significant interaction between the two (F(1, 11) = 6.82, p = 0.024). Tukey’s post-hoc comparisons indicated a significant decrease in DSA from initial to late Adapt 1 (t(11) = −4.16, p = 0.002) and initial to late Adapt 2 (t(11) = 2.21, p = 0.049). These observations underscore the ability of able-bodied individuals to bilaterally adapt spatial and temporal interlimb coordination in response to the imposed belt speed perturbations.

### Motor Learning: Net Metabolic Power

Given that spatiotemporal adaptations are paralleled by reductions in metabolic cost during split-belt walking^12,22^, we quantified concurrent changes in net metabolic power (W/kg) utilizing open-circuit spirometry. To account for the time lag of expired air reaching the mixing chamber, we quantified changes in ‘early’ and ‘late’ net metabolic power during the second and last minute of each condition, respectively (Fig. 3D)^22^.

An ANOVA detected significant main effects of condition (F(1, 11) = 9.76, p = 0.010) and an interaction effect between condition and learning phase (F(1, 11) = 17.60, p = 0.001). Tukey’s post-hoc comparisons identified a significant decrease in net metabolic power from early to late Adapt 1 (t(11) = 3.90, p = 0.002) and from early to late Adapt 2 (t(11) = −2.47, p = 0.031). Mean net metabolic power decreased from 6.09 W/kg during early Adapt 1 (SE = 0.44) to 5.73 W/kg during late Adapt 1 (SE = 0.38). Thus, as participants adapted their coordination, they simultaneously improved their metabolic efficiency.

### Lower Limb Excitability Predicts Motor Learning: Spatiotemporal Asymmetry

**Figure 4.**
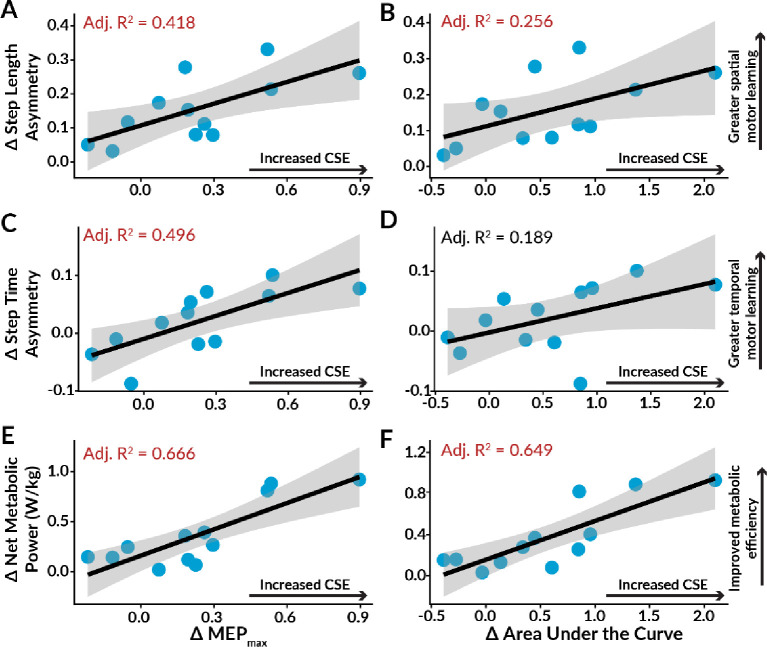
Linear regressions among TMS indices of AIH-induced enhancements in corticospinal excitability and their correlation with motor learning outcomes. The red text indicates statistically significant adjusted R^2^ values (p < 0.05), while the black text signifies insignificant values. **A.** We observed a significant positive correlation between increased maximum motor-evoked potential amplitude (MEP_max_) post-AIH and improvements in step length asymmetry during Adapt 1. **B.** The association between elevated area under the recruitment curve post-AIH and enhanced step length asymmetry during motor learning approached significance (p = 0.053). **C.** We identified a significant correlation between elevated MEP_max_ post-AIH and augmented step time asymmetry during Adapt 1. **D.** Correlations between increased area under the recruitment curve post-AIH and greater step time asymmetry during motor learning approached significance (p = 0.088). **E.** Elevated MEP_max_ after AIH exhibited a positive correlation with decreased net metabolic power during motor learning. **F.** Heightened area under the recruitment curve post-AIH positively correlated with reduced net metabolic power during Adapt 1. Individual data points are represented in blue, and the 95% confidence interval is illustrated by the grey-shaded area.

We performed linear regression analyses and calculated the adjusted R^2^ values to evaluate how well Δ MEP_max_, Δ area under the recruitment curve, Δ peak slope, and Δ RMT predicted the magnitude of motor learning (Fig. 4, Table 1). We observed a significant positive correlation between increased MEP_max_ after AIH and reduced SLA during motor learning (Adjusted R^2^ = 0.418, p = 0.014). Moreover, a positive trend emerged between elevated area under the recruitment curve after AIH and decreased SLA during motor learning (Adjusted R^2^ = 0.256, p = 0.053). Stepwise linear regression identified both Δ MEP_max_ and Δ peak slope as the primary predictors of improved SLA during motor learning (Δ SLA = 0.097 + 0.420 * Δ MEP_max_ − 0.423 * Δ peak slope, AIC = −65.29, model p = 0.039, MEP_max_ p = 0.032, peak slope p = 0.0395), together explaining 56.72% of the variance.

For motor learning of temporal adaptation, we observed a significant positive correlation between increased MEP_max_ after AIH and higher STA during motor learning (Adjusted R^2^ = 0.496, p = 0.006). Furthermore, the correlation between increased area under the recruitment curve after AIH and heightened STA approached significance (Adjusted R^2^ = 0.189, p = 0.088), as did the correlation between increased peak slope post-AIH and more positive STA (Adjusted R^2^ = 0.233, p = 0.064). Utilizing stepwise linear regression, Δ MEP_max_, Δ area under the recruitment curve, and Δ RMT emerged as the best predictors of changes in STA during motor learning (Δ STA = 0.015 + 0.245 * Δ MEP_max_ − 0.071 * Δ area under the recruitment curve + 0.008 * Δ RMT, AIC = −76.15, model p = 0.055, MEP_max_ p = 0.0297, area under the recruitment curve p = 0.120, RMT p = 0.194), explaining 51.82% of the variance. Conversely, none of the TMS indices showed a significant correlation with decreased DSA during motor learning (model p = 0.967). In summary, TMS indices reflecting changes in CSE after AIH significantly predicted the magnitude of motor learning observed for both SLA and STA.

### Lower Limb Excitability Predicts Motor Learning: Net Metabolic Power

Interestingly, net metabolic power significantly correlated with Δ MEP_max_ (Adjusted R^2^ = 0.666, p = 0.001), Δ area under the recruitment curve (Adjusted R^2^ = 0.649, p = 0.001), and Δ peak slope (Adjusted R^2^ = 0.313, p = 0.034), while Δ RMT approached significance (Adjusted R^2^ = 0.204, p = 0.079). Stepwise linear regression identified Δ MEP_max_ as the most significant predictor of reduced net metabolic power during motor learning (Δ net metabolic power = 0.161 + 0.875 * Δ MEP_max_, AIC = −38.35, model p = 0.021). Together, these results suggest that TMS indices of CSE were significant predictors of the decrease in net metabolic power that accompanies spatiotemporal adaptation during motor learning.

### Motor Savings: Spatiotemporal Asymmetry

**Figure 5.**
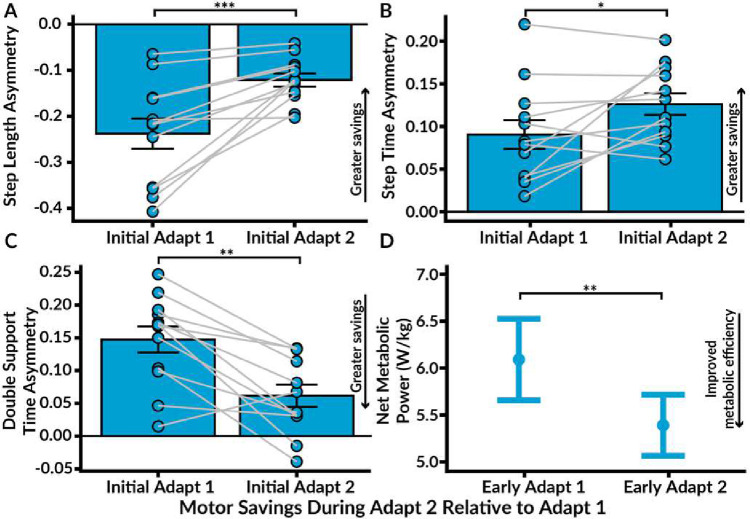
Motor Savings. We characterized motor savings by measuring the degree of spatiotemporal adaptation from initial Adapt 1 to initial Adapt 2 and the extent of metabolic adaptation from early Adapt 1 to early Adapt 2. **A.** Savings of step length asymmetry were evidenced by significantly lower asymmetry during initial Adapt 2 relative to initial Adapt 1. **B.** Step time asymmetry was significantly elevated during initial Adapt 2 compared to initial Adapt 1. **C.** Savings of double support time asymmetry were observed, as asymmetry was significantly lower during initial Adapt 2 compared to initial Adapt 1. **D.** Savings of improved metabolic efficiency were also shown by lower net metabolic power during Adapt 2 compared to Adapt 1. The bars represent standard error. Significance levels are denoted as *** p < 0.001, ** p < 0.01, and * p < 0.05.

We characterized motor savings by quantifying the magnitude of spatiotemporal adaptation retained during initial Adapt 2 compared to initial Adapt 1 (Fig. 5A–C)^24^. We observed significant savings of spatial symmetry, as evidenced by more symmetric step lengths during initial Adapt 2 relative to initial Adapt 1 (t(11) = −4.46, p < 0.001) and during late Adapt 2 relative to late Adapt 1 (t(11) = −4.70, p < 0.001). Additionally, we found significant savings of both STA and DSA. STA was significantly higher during initial Adapt 2 compared to initial Adapt 1 (t(11) = −2.35, p = 0.039), as well as from late Adapt 2 compared to late Adapt 1 (t(11) = −2.41, p = 0.035). For DSA, Tukey’s post-hoc comparisons identified significantly lower asymmetry during initial Adapt 2 compared to initial Adapt 1 (t(11) = 4.43, p = 0.001) and from late Adapt 2 compared to late Adapt 1 (t(11) = 3.02, p = 0.012). Therefore, participants displayed motor savings of both spatial and temporal parameters of adaptation.

### Motor Savings: Net Metabolic Power

We assessed savings of metabolic adaptation by comparing net metabolic power during early Adapt 2 to early Adapt 1 (Fig. 5D). Tukey’s pairwise comparisons indicated significantly lower net metabolic power during early Adapt 2 compared to early Adapt 1 (t(11) = 3.75, p = 0.003), demonstrating that participants retained improved metabolic efficiency. The mean net metabolic power during early Adapt 2 was 5.39 W/kg (SE = 0.33), with a 0.7 W/kg reduction compared to early Adapt 1.

### Lower Limb Excitability Predicts Motor Savings: Spatiotemporal Asymmetry

**Figure 6.**
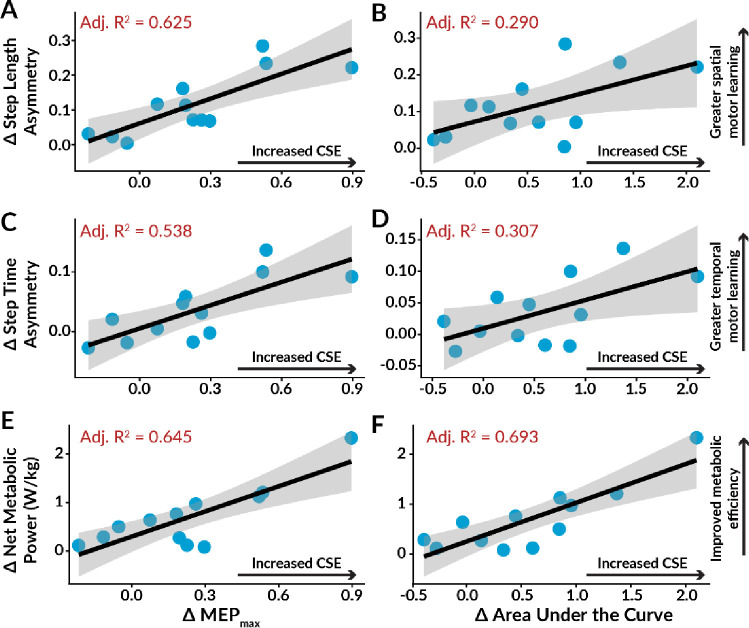
Linear regressions among indices of AIH-induced enhancements in corticospinal excitability and their correlation with motor savings outcomes. The red text indicates statistically significant adjusted R^2^ values (p < 0.05), while the black text signifies insignificant values. **A.** We identified a significant correlation between increased maximum motor-evoked potential amplitude (MEP_max_) after AIH and savings of lower step length asymmetry. **B.** Increased area under the recruitment curve post-AIH exhibited a significant correlation with the savings of lower step length asymmetry. **C.** We observed a significant correlation between heightened MEP_max_ post-AIH and the savings of higher step time asymmetry. **D.** Increased area under the recruitment curve post-AIH positively correlated with the savings of higher step time asymmetry. **E.** There was a significant correlation between elevated MEP_max_ after AIH and the savings of lower net metabolic power. **F.** We found a positive correlation between a larger area under the recruitment curve post-AIH and the savings of lower net metabolic power. Individual data points are shown in blue, and the 95% confidence interval is represented by the grey-shaded area.

To evaluate how well Δ MEP_max_, Δ area under the recruitment curve, Δ peak slope, and Δ RMT explained the variance in motor savings outcomes, we conducted linear regression analyses and computed adjusted R^2^ values (Fig. 6, Table 2).

A significant positive correlation was observed between elevated MEP_max_ following AIH and the savings of lower SLA (Adjusted R^2^ = 0.625, p = 0.001). Furthermore, the increased area under the recruitment curve positively correlated with the savings of lower SLA (Adjusted R^2^ = 0.290, p = 0.041). Stepwise linear regression identified Δ MEP_max_ and Δ peak slope as the two best predictors of SLA savings (Δ SLA = 0.053 + 0.416 * Δ MEP_max_ − 0.366 * Δ peak slope, AIC = −75.05, model p = 0.005, MEP_max_ p = 0.002, slope p = 0.0423), explaining 77.33% of the variance.

Linear regression analyses also revealed a significant positive correlation between increased MEP_max_ after AIH and the savings of elevated STA (Adjusted R^2^ = 0.538, p = 0.004). Additionally, we observed a significant positive correlation between the elevated area under the recruitment curve and the savings of higher STA (Adjusted R^2^ = 0.307, p = 0.036). Stepwise linear regression indicated that both Δ MEP_max_ and Δ peak slope were the primary predictors of STA savings (Δ STA = 0.002 + 0.203 * Δ MEP_max_ − 0.152 * Δ peak slope, AIC = −79.24, model p = 0.065, MEP_max_ p = 0.039, peak slope p = 0.261).

Both the Δ MEP_max_ and Δ peak slope explained 49.03% of the variance in STA savings. However, no significant correlations were observed between any of the TMS indices and the savings of lower DSA (model p = 0.468). These results suggest that TMS indices of CSE were strong predictors of the degree of motor savings observed for both SLA and STA.

### Lower Limb Excitability Predicts Motor Savings: Net Metabolic Power

Net metabolic power exhibited a robust positive correlation with Δ MEP_max_ (Adjusted R^2^ = 0.645, p = 0.001), Δ the area under the recruitment curve (Adjusted R^2^ = 0.693, p < 0.001), and Δ peak slope (Adjusted R^2^ = 0.406, p = 0.015), with Δ RMT approaching significance (p = 0.065). Stepwise linear regression analysis identified Δ area under the recruitment curve as the best predictor of net metabolic power savings (Δ net metabolic power = 0.251 + 0.776 * Δ area under the recruitment curve, AIC = −22.76, model p = 0.024). Overall, we found that TMS indices representing changes in CSE post-AIH significantly predicted the savings of lower net metabolic power upon subsequent split-belt walking exposures.

### Spatiotemporal Asymmetry Predicts Net Metabolic Power

Both Δ SLA and Δ STA significantly correlated with Δ net metabolic power during motor learning (Δ SLA Adjusted R^2^ = 0.452, p = 0.010; Δ STA Adjusted R^2^ = 0.396, p = 0.017) and motor savings (Δ SLA Adjusted R^2^ = 0.461, p = 0.009; Δ STA Adjusted R^2^ = 0.468, p = 0.008). In contrast, Δ DSA did not correlate with Δ net metabolic power (motor learning p = 0.734; motor savings p = 0.580). While changes in spatiotemporal asymmetry significantly predicted improvements in metabolic efficiency (Supplementary Fig. S1), changes in MEP_max_ and the area under the recruitment curve explained a greater portion of the variance in net metabolic power.

### Retrospective Comparison of Learning Between a Mild vs. Large Perturbation

To better understand how perturbation size affects motor learning and motor savings after AIH, we retrospectively compared the degree of spatiotemporal and metabolic adaptation between a large 1:2 belt speed ratio and a milder 1:1.5 belt speed ratio reported in Bogard et *al*. 2023 (See Supplementary Fig. S2). The results of the statistical tests are summarized in Supplementary Table S3. Briefly, Tukey’s post-hoc analyses indicated that the large perturbation group achieved more symmetrical step lengths during both motor learning and motor savings compared to the mild perturbation group. The average decrease in SLA for the large perturbation group was 0.09 lower during motor learning (SE = 0.03) and 0.07 lower during motor savings (SE = 0.03) than the mild perturbation group. The large perturbation group also demonstrated greater motor savings of STA, maintaining an average STA that was 0.04 higher than the mild perturbation group (SE = 0.02). Both the mild and large perturbation groups displayed similar magnitudes of motor learning and motor savings of DSA. The large perturbation group exhibited greater savings of improved metabolic efficiency, with net metabolic power that was an average of 0.57 W/kg lower than the mild perturbation group (SE = 0.20). Although we did not find a significant group difference in net metabolic power during motor learning, the large perturbation group averaged 0.35 W/kg lower than the mild perturbation group (SE = 0.20). Overall, the larger perturbation led to greater motor learning of SLA and more prominent motor savings of SLA, STA, and metabolic efficiency.

## Discussion

Our results provide the first evidence that repetitive AIH amplifies excitability to the lower limb, supporting our primary hypothesis. Significantly, this enhanced excitability post-AIH is associated with both improved motor learning and motor savings, confirming our secondary hypothesis. Furthermore, our study demonstrates a novel association between elevated lower limb excitability and greater metabolic efficiency. Together, these results indicate that both changes in excitability and reductions in net metabolic power are markers of AIH-mediated neuroplasticity.

### AIH Increases Lower Limb Excitability

Our results advance the understanding of AIH-induced neuroplasticity beyond the upper limb by demonstrating that repetitive AIH increases TA MEP_max_, area under the recruitment curve, and MEP amplitudes at both 120% and 140% of RMT. Additionally, our study did not detect any concurrent changes in the maximal voluntary contraction after AIH, aligning with prior research^26^. This suggests that changes in maximal voluntary contraction may not parallel changes in descending and spinal excitability in able-bodied individuals. While the composite nature of MEPs precludes the differentiation of the specific contributions of intracortical and transcortical networks to behavioral outcomes^27^, our collective findings indicate that AIH increases net motoneuronal excitability. Indeed, prior research on the upper limb has shown increases in both CSE and spike-timing-dependent plasticity after a single AIH dose, whereas measures of intracortical inhibition and facilitation remained unchanged^9^. Although we did not examine spinal excitability, Finn et *al.* (2022) observed a lower threshold for eliciting the soleus H-reflex after AIH, indicating enhanced efficacy of the Ia-afferent-motoneuronal pathway. Together with our observations suggest that AIH enhances motoneuronal excitability.

It is important to acknowledge that inconsistent findings have been reported. Discrepancies may be partially attributed to variations in AIH dosing. For instance, compared to Christiansen et *al*. (2018) and our study, Finn et *al*. (2022) only observed increased CSE at discrete time points while utilizing a more conservative desaturation threshold of 75% and omitting the remaining hypoxic interval. In contrast, Radia et *al.* (2022) implemented a longer 3-minute hypoxic interval and did not detect changes in CSE. Notably, we observed significant increases in CSE with multiple-day AIH administration, utilizing dosing intervals that conferred improvements in walking performance after iSCI^2,3^. Given the distinct cortical representations^28^ and varying motor thresholds of upper and lower limb muscles^29^, it is also plausible that AIH may not produce the same magnitude of change across all motoneuron pools. Nevertheless, our findings of increased TA excitation after repetitive AIH suggest potential applications in neurorehabilitation to promote adaptive neuroplasticity.

### Lower Limb Excitability Predicts Motor Learning and Motor Savings

To our knowledge, this is the first study to examine the functional significance of AIH-induced enhancements in CSE in humans. We demonstrate that the degree of CSE enhancement post-AIH predicts gains in motor learning, as evidenced by a positive correlation between increased MEP_max_ and greater spatiotemporal adaptation. Additionally, increased MEP_max_ and area under the recruitment curve post-AIH both correlate with larger savings of spatiotemporal adaptation. We argue for the interpretation that enhanced CSE is not only a marker of AIH-mediated neuroplasticity but also supports the processes that underlie the acquisition and savings of new motor skills. This is supported by preceding investigations that have linked CSE to improvements in both motor learning^30,31^ and motor savings^15,32^.

The argument can be made that if heightened CSE supports motor learning processes, individuals who do not exhibit an increase in CSE after AIH may not learn the task. However, able-bodied individuals can still acquire new motor skills without changes^22,24^ or even with decreases in CSE^33^. Indeed, participants with no changes or decreases in CSE still successfully adapted spatiotemporal coordination, albeit to a lesser extent (Fig. 5). It is also possible that increased motor learning after AIH^12^ further drives increases in CSE. However, it is unlikely that improvements in motor learning alone facilitate increases in CSE as motor training paradigms show that elevated CSE only persists with additional task challenges^17,34^. Moreover, we distinctively evaluated changes in CSE before assessing motor learning within a conservative 75-minute window. While we did not assess longitudinal changes in CSE, previous research indicates CSE remains elevated for up to 75 minutes after a single AIH dose^9^. Therefore, our results suggest that even transient increases in CSE after repetitive AIH predict improvements in both motor learning and motor savings.

Of particular interest is that we characterized improvements in motor learning and motor savings without any concurrent motor training paradigm. Previous work in humans demonstrated that AIH paired with task-specific motor training further amplifies walking performance after iSCI compared to AIH or training alone^2,3^. Motor training may further potentiate changes in AIH-induced neuroplasticity as longitudinal human studies have shown that progressively challenging motor training enhances both motor learning and CSE^17,35^.

### Enhanced Excitability Predicts Greater Reductions in Net Metabolic Power

Our findings reveal an unprecedented positive correlation between increased CSE and improved metabolic efficiency for both motor learning and motor savings. Specifically, increased MEP_max_, area under the recruitment curve, and peak slope all positively correlate with reduced net metabolic power. Therefore, improved metabolic efficiency could serve as a marker of AIH-induced neuroplasticity. This interpretation is supported by our previous study, where the AIH group displayed lower net metabolic power at the start of the novel task and uniquely demonstrated significant savings of improved metabolic efficiency^12^.

Considering that improved metabolic efficiency is a feature of gains in motor learning^36^, it is plausible that the reductions in net metabolic power are primarily driven by improvements in spatiotemporal adaption. However, we observed that most of the TMS indices explained more variance in net metabolic power than spatiotemporal asymmetry. This indicates that metabolic efficiency cannot be explained by changes in biomechanics alone and that net metabolic power provides an additional marker of AIH-mediated neuroplasticity.

### Potential Mechanisms of Increased CSE

Evidence from rodent models suggests that BDNF-dependent mechanisms play a central role in AIH-mediated neuroplasticity^4–6^. Therefore, it is plausible that BDNF may serve as the intermediary link between AIH-induced increments in CSE and enhancements in motor learning. Prior investigations in exercise-dependent increases in BDNF^37^ substantiate this interpretation by showing concomitant increases in CSE and motor learning^14^. Conversely, individuals with a BDNF val66met polymorphism, known to decrease activity-dependent BDNF secretion, do not exhibit a significant change in CSE following a complex motor task^38^, whereas increasing CSE through motor cortex stimulation enhances motor learning^13^.

While our study did not assess the involvement of BDNF, its established role in contributing to energy efficiency^39^ by promoting synaptic plasticity^40^ and corticospinal synaptogenesis^41^ could suggest that heightened CSE after AIH may facilitate BDNF-mediated energy efficiency. Critical for this interpretation, the AIH-dependent upregulation of BDNF has been shown to strengthen excitatory glutamatergic synapses in rodents with iSCI^42^. Importantly, rodents with increased BDNF signaling demonstrate increased excitatory neurotransmission and plasticity^43^. In humans, genetic disruption of BDNF impairs the formation of excitatory glutamatergic synapses^44^. Future studies are needed to clarify the role of BDNF in regulating metabolic efficiency in humans.

### Larger Perturbations May Promote Better Motor Savings

While our previous study revealed significant improvements in motor learning and savings after AIH at a mild perturbation^12^, it remains unclear whether AIH-induced enhancements in CSE differentially facilitate motor learning and savings with higher levels of difficulty. Prior work found that continuously increasing the task difficulty drives persistent increases in CSE^17^. Additionally, earlier studies have demonstrated that greater challenges, such as larger split-belt speed perturbations, result in enhanced motor learning and savings^24^. Consistent with earlier findings^24^, we observed greater motor learning and savings of SLA with the large perturbation. Notably, we also observed greater savings of higher STA and improved metabolic efficiency with the large perturbation. Our findings suggest that AIH-induced increases in CSE may exert distinct effects as the challenge increases, with greater challenges leading to more substantial enhancements in both motor learning and metabolic efficiency. Tailoring task difficulty has the potential to optimize rehabilitation strategies post-AIH.

In conclusion, our study not only uncovers the positive effects of repetitive AIH on lower limb excitability, but also establishes its critical role in predicting motor learning, motor savings, and metabolic efficiency. Optimizing motor learning to drive gains in motor function after neurological injury necessitates a closer examination of the interplay between AIH-induced corticospinal plasticity and the structure of motor training during rehabilitation.

## Methods

### Participants

Thirteen able-bodied subjects between the ages of 18 to 65 participated in the study at the University of Colorado, Boulder (Females = 7; Mean age = 23.4 ± 1.8 years; Mean height = 173.6 ± 9.4 cm; Mean weight = 72.6 ± 17.0 kg). Demographic characteristics are summarized in Supplementary Table S4. An a priori analysis determined that a sample size of 12 participants would be sufficient to detect significant difference in corticospinal excitability (CSE) across 2 time points (e.g., pre-AIH and post-AIH) with >80% power. Exclusion criteria comprised a history of cardiovascular disease, pulmonary complications, pain, syncope, sensitivity to altitude, or currently pregnant or undergoing physical therapy. All participants provided written informed consent from the Colorado Multiple Institutional Review Board (COMIRB #20–0689) before participation. The study adhered to the principles of the Declaration of Helsinki, and registration was completed at clinicaltrials.gov (NCT05341466; 22/04/2022).

### Experimental Design

Subjects visited the laboratory for five consecutive days to participate in experiments. On the first day, we assessed baseline TA excitability using TMS before administering the first dose of AIH (‘pre-TMS’). Following the pre-TMS session, all thirteen participants received AIH for five consecutive days at the same time each day. Participants were not informed of whether they received AIH or normoxic air. Prior evidence indicates that participants cannot distinguish between AIH vs. SHAM exposures (i.e., normoxic air)^45^. A single AIH dose entailed 15 cycles, each comprised of 90-second intervals of breathing low-oxygen air (9% ± 2% O_2_) alternated with 60-second intervals of breathing ambient air (21% ± 2% O_2_)^3^. During AIH, two trained operators manually regulated the supply of low-oxygen air by connecting a hose to a non-rebreather mask^46^. We recorded oxygen saturation (SpO_2_) and heart rate (HR) at 1 Hz while blood pressure (BP) was measured every five cycles (Masimo; Irvine, CA). We paused the hypoxic interval if SpO_2_ fell below 70%, HR exceeded 160 bpm or systolic BP went above 160 mmHg and resumed once SpO_2_ returned above 80%, HR dropped below 140 bpm, and systolic BP decreased below 140 mmHg. The experiment terminated if the participant reported pain, dizziness, diaphoresis, tinnitus, or blurred vision. On the last day, participants underwent a second TMS session within 15 minutes after completing their final dose of AIH (‘post-TMS’). The split-belt walking task was performed following the second TMS session to ensure that motor learning did not influence CSE.

### Transcranial Magnetic Stimulation Protocol

To examine alterations in resting CSE, we utilized monophasic, single-pulse TMS (DuoMAG MP-Dual Magnetic Stimulator; Deymed, Czechia) to target the leg motor area of the primary motor cortex^47^. We positioned an insulated, double-cone coil (DuoMAG Butterfly V-Shaped Coil; Magstim, UK) 0–2 cm posterior to the vertex to evoke MEPs in the TA muscle, targeting the region that obtained the lowest threshold and shortest latency response^48^ recorded via surface electromyography (EMG) (Bipolar Ag/AgCl, spaced 22mm apart, Noraxon Inc., USA). Notably, resting TA MEPs are highly reproducible^49^ and the TA requires a lower stimulus intensity than other muscles^50^. Prior studies have established a close link between corticospinal drive and TA excitability^51^. Additionally, enhancing TA excitability holds clinical significance as it would improve foot clearance during walking after iSCI^52^.

We utilized a generic brain MRI scaled to anatomical references and an NDI Polaris Vicra camera for real-time navigation of the TMS coil (Visor2, ANT-NeuroNav, Netherlands). The hotspot was defined as the stimulation vector that elicited 4 MEPs ≥ 50 μV out of 7 trials^53^. The resting motor threshold (RMT) wsas determined as the lowest stimulus intensity to elicit at least 4/7 MEPs ≥ 50 μV over the hotspot. We used 7 trials as a conservative intermediary between the commonly used 5-trial criterion^54^ and the 10-trial criterion^55^.

Recruitment curves were generated by plotting the peak-to-peak amplitudes of the TA MEPs against their corresponding stimulus intensities. To compare changes in CSE across participants and testing sessions pre- to post-AIH, we normalized the recruitment curves to the RMT during each respective testing session^11^. Thus, the recruitment curve sampling involved a pseudo-randomized sequence of stimulations ranging from 90% to 140% of the RMT^56,57^, with 6 pulses applied per intensity (Signal; CED, USA)^58^. We delivered TMS stimuli in 15-second intervals with a 15% time variation to prevent synaptic fatigue and minimize MEP variability^59^.

### Split-Belt Walking Protocol

During the split-belt walking protocol, participants learned to walk on treadmill belts set at different speeds (D-flow v3.34.3; Motek, Netherlands). We secured participants into a passive safety harness and instructed them to refrain from using the handrails. To prevent inadvertent stepping onto the contralateral treadmill belt, participants were permitted to observe their feet in a mirror^12^.

The split-belt walking protocol comprised of four walking conditions: (1) ‘Baseline’, with matched belt speeds set at 1 m/s for 300 strides, (2) ‘Adapt 1’, involving a 2:1 split-belt speed ratio for 300 strides, (3) ‘Washout’, with tied-belts set at 1 m/s for 350 strides, and (4) ‘Adapt 2’, replicating the 2:1 split-belt speed ratio for 300 strides^24^. The perturbed belt was randomized^60^. Importantly, participants remained unaware that the leg perturbed during the split-belt walking protocol corresponded to the same leg used for recording surface EMG during the TMS protocol. We increased the belt speed 15–30 strides into the adaptation condition to blind the subjects to the timing^12^.

### Data Collection

#### Electromyography.

We placed bipolar Ag/AgCl electrodes, spaced 22 mm apart, over the TA, one-third of the distance from the fibular head to the lateral malleolus, and a ground electrode over the patella (Noraxon Inc., USA)^61^. To obtain low impedance (< 5 kΩ), we first shaved and cleaned the skin with an isopropyl alcohol wipe^62^. EMG signals were bandpass filtered within a range of 20–1000 Hz, amplified by a gain 100 (CED 1902), sampled at 10 kHz (CED 1401), and displayed in real-time using Signal software (CED, USA).

#### Maximal voluntary contraction.

Before the first AIH dose and after the fifth and final AIH dose, participants were instructed to generate three, 5-second maximal dorsiflexion contractions against a resistive load, with a minimum 1-minute rest between trials^63^. We directed participants to contract their TA as forcefully and rapidly as possible without engaging their thigh muscles^64^. If the maximal peak-to-peak amplitude varied by more than 10%, the participant performed additional contractions^65^. We defined the maximal voluntary contraction as the highest value obtained.

#### Kinematics.

A 10-camera motion system captured lower limb kinematic data at a sampling frequency of 100 Hz (Vicon Nexus v2.8.1; Vicon Motion Systems, UK). We bilaterally placed reflective markers over the shank and thigh, as well as on the anterior and posterior superior iliac spines, iliac crests, greater trochanters, medial and lateral femoral epicondyles, medial and lateral malleoli, calcanei, and first and fifth metatarsal heads^12^. We analyzed kinematic data using custom pipelines in Visual3D (v2021.11.3; C-motion Inc., MD) and MATLAB (R2023b; MathWorks, Inc., US).

#### Kinetics.

Three-dimensional ground reaction forces (GRF) were collected at a sampling frequency of 1,000 Hz (D-flow v3.34.3; Motek, Netherlands) and low-pass filtered with a fourth-order Butterworth filter at a cutoff frequency of 20 Hz^66^. We used GRFs to validate gait events.

#### Expired gas analysis.

We measured the rate of oxygen consumption (VO_2_) and carbon dioxide production (VCO_2_) using open circuit spirometry (TrueOne 2400; ParvoMedics Inc., UT). Resting metabolic rate was estimated based on the average VO_2_ and VCO_2_ of the last 2 minutes of a 5-minute standing trial and was subtracted from the subsequent net metabolic power measured during walking^22^. The mean resting metabolic rate was 1.42 W/kg (SE = 0.05). Respiratory exchange ratios (RER; VCO_2_/VO_2_) below 1 indicated predominant utilization of aerobic pathways^67^.

### Data Analysis

#### Maximum Motor Evoked Potential.

The MEP onset was identified using custom configurations in Signal (CED, USA) as the first frame at which the peak-to-peak amplitude exceeded three standard deviations of the background EMG^49^ recorded 200 ms preceding the TMS stimuli^23^. To account for the pre-stimulus baseline TA activation, we subtracted the mean background EMG from all MEPs^68^. We averaged the peak-to-peak MEP amplitudes across all thirteen participants and quantified the change in MEP_max_ pre-AIH and post-AIH (Δ MEP_max_).

#### Area Under the Recruitment Curve.

We calculated the area under the recruitment curve both pre- and post-AIH (Δ area under the recruitment curve) using the trapezoidal method of area estimation. The area under the recruitment curve provides a crude measure of overall net excitability from 90% to 140% of RMT^69^.

#### Recruitment Curve Slope.

The recruitment curve slope was determined by fitting a Boltzmann distribution in MATLAB using the Levenberg-Marquardt algorithm, as shown in Equation 1^70^. The Boltzmann equation is a function of stimulus intensity (s) and response amplitude (MEP), where $MEP_{max}$ is the maximum averaged response from the recruitment curve, $s_{50}$ is the stimulus intensity required to produce a response with half the amplitude of $MEP_{max}$, and k is the slope parameter^71^.


(Equation 1) $$MEP(s)=\frac{MEPmax}{1+e^{\left(S_{50}-s\right)/k}}$$

Peak slope, the inverse of the slope parameter, represents the rate of the MEP increase relative to $MEP_{max}$ and is used to quantify the change in the gain of excitability^58^. We calculated alterations in peak slope pre-AIH and post-AIH (Δ peak slope).

#### Resting Motor Threshold.

RMT represents the motor threshold required to excite the TA at rest^53^. We compared the RMT before and after AIH (Δ RMT).

#### Spatiotemporal Asymmetry.

We identified heel-strike and toe-off gait events for each stride using a vertical GRF detection threshold of 30 N^72^. These gait events were utilized to calculate step length asymmetry (SLA), step time asymmetry (STA), and double support time asymmetry (DSA). Step lengths and step times were determined by the fore-aft contralateral calcanei markers at heel-strike, while double support times were defined as the duration between the leading limb’s heel-strike and contralateral toe-off^73^. We computed asymmetry values using Equation 2^74^, where values of 0 indicate perfect symmetry.


(Equation 2) $$\text{Asymmetry}=\frac{\text{Fast}\;\text{leg}\;-\;\text{Slow}\;\text{leg}}{\text{Fast}\;\text{leg}\;+\;\text{Slow}\;\text{leg}}$$

The phases ‘initial learning’ and ‘late learning’ were defined as the first five strides after the speed perturbation and the final twenty strides, respectively^24^. We assessed motor learning by examining the magnitude of spatiotemporal adaptation from initial Adapt 1 to late Adapt 1 and motor savings by the extent of adaptation from initial Adapt 1 to initial Adapt 2. Enhanced motor learning and motor savings were characterized by reductions in SLA and DSA towards symmetry values of 0, coupled with augmentations in STA towards positive asymmetry. This is supported by extensive research demonstrating these adaptation patterns in able-bodied individuals^25,75,76^. Given evidence of a distinct mode-dependency in the neural control of split-belt walking compared to split-belt running^77^, we excluded one participant from gait analyses due to utilizing a running strategy with a distinct aerial phase during both Adapt 1 and Adapt 2.

#### Net Metabolic Power.

We quantified metabolic power using a standard regression equation^78^. The mean resting metabolic rate was subtracted from metabolic power and then normalized to body mass to compute net metabolic power (W/kg)^79^. We omitted the first minute of each condition to account for the time lag of the expired gas reaching the mixing chamber^12^. Thus, we defined ‘early’ and ‘late’ net metabolic power as the second and last minute of each condition, respectively^22^. We characterized the extent of metabolic adaptation during motor learning by assessing the difference in net metabolic power from early Adapt 1 to late Adapt 1. Additionally, we characterized metabolic savings by comparing early Adapt 1 and early Adapt 2.

#### Retrospective Comparisons.

To gain deeper insights into how perturbation size affects AIH-induced enhancements in motor learning and motor savings, we conducted a retrospective analysis comparing the magnitude of spatiotemporal and metabolic adaptation between a large 1:2 belt speed ratio and a milder 1:1.5 belt speed ratio. The mild perturbation group comprised a subset of participants from the AIH group reported in Bogard et *al*. (2023). Converging evidence suggests that increasing task difficulty can improve subsequent motor learning^24,35^ and potentiate cortical plasticity^17^. Thus, the primary objective of this analysis was to determine whether AIH further enhances motor learning and motor savings under greater challenges, considering its demonstrated efficacy even under mild challenges^12^. Determining specific split-belt walking parameters that optimize motor learning and motor savings will clarify our understanding of the effects of AIH on locomotor adaptation.

### Statistical Analysis

Statistical analyses of the experimental data were conducted in R Studio (v2021.09.0) with a significance level set at p < 0.05. Group data are presented as mean ± standard error (SE). We evaluated normality using the Shapiro-Wilks test, multicollinearity using the variance of inflation factor, and sphericity using Mauchly’s Test.

#### Background EMG.

To evaluate the consistency of the pre-stimulus background EMG between pre-AIH and post-AIH TMS sessions, we conducted a paired t-test.

#### TA excitability.

We assessed alterations in TA excitability pre-AIH and post-AIH using paired t-tests for normally distributed data and Wilcoxon signed-rank tests when normality assumptions were not met. Specifically, we used paired t-tests to analyze changes in the maximal voluntary contraction, and Wilcoxon signed-rank tests to assess changes in MEP_max_, area under the recruitment curve, peak slope, and RMT. In addition, we conducted a repeated measures ANOVA with the Greenhouse-Geisser degrees of freedom correction to compare the peak-to-peak MEP amplitude pre-AIH and post-AIH at the stimulus intensities of 100% RMT, 120% RMT, and 140% RMT, with the within-factors of ‘stimulus intensity’ (100%, 120%, and 140% RMT) and ‘timepoint’ (pre-AIH and post-AIH). We conducted post hoc analyses utilizing Tukey’s honest significant difference method to correct for multiple comparisons.

#### Adaptation.

We conducted a two-way ANOVA to investigate the main effects of the within-factors ‘condition’ and ‘learning phase’ on spatiotemporal asymmetry (SLA, STA, DSA) and net metabolic power. We treated the subject identification number as a random factor to acknowledge that the same participants were measured across different levels of the within-subject factors, contributing to a repeated measures design. Assumptions of sphericity were not violated for any of the dependent variables; thus, corrections were not applied. Subsequently, we conducted two Tukey’s post-hoc tests for each ANOVA to explore any potential significant pairwise comparisons. We used Tukey’s pairwise comparisons to analyze motor learning of spatiotemporal symmetry between initial Adapt 1-late Adapt 1 and initial Adapt 2-late Adapt 2. Additionally, comparisons were made between early Adapt 1-late Adapt 1 to evaluate metabolic adaptation. We also performed Tukey’s pairwise comparisons between initial Adapt 1-initial Adapt 2 and late Adapt 1-late Adapt 2 to assess savings of spatiotemporal adaptation, as well as between early Adapt 1-early Adapt 2 to examine savings of metabolic efficiency.

#### Correlations.

We performed linear regression analyses between TMS indices of CSE and motor learning outcomes, as well as between TMS indices of CSE and motor savings outcomes. To compare how well TMS indices of CSE explained the variance in net metabolic power compared to parameters of spatiotemporal asymmetry, we also performed linear regression analyses with Δ SLA, Δ STA, and Δ DSA as the predictors and Δ net metabolic power as the outcome variable. We calculated the adjusted R^2^ values for each linear regression to measure the proportion of the variance in behavior explained by changes in CSE. In addition, we used stepwise linear regression models to iteratively eliminate insignificant predictor variables. For each model, the predictor variables were Δ MEP_max_, Δ area under the recruitment curve, Δ peak slope, and Δ RMT, and the dependent variables were the motor learning and motor savings outcomes (Δ SLA, Δ STA, Δ DSA, and Δ net metabolic power). The final stepwise linear regression model was determined based on achieving the lowest Akaike information criterion (AIC) and highest adjusted R^2^ value.

#### Retrospective comparisons.

We compared motor learning and motor savings between a mild and a large perturbation. To address differences in sample size, we utilized linear mixed models, incorporating Satterthwaite’s method for estimating degrees of freedom. Subsequently, we used an ANOVA to assess significance, with perturbation size as the between-group factor (1:1.5 belt speed ratio and 1:2 belt speed ratio) and adaptation period as the within-group factor (Δ motor learning and Δ motor savings). Significant ANOVAs were followed by Tukey’s post-hoc analysis to adjust for multiple comparisons.

## Supplementary Material

Supplement 1

## Figures and Tables

**Table 1.** Summary of the linear regression analyses performed between indices of AIH-induced enhancements in corticospinal excitability and motor learning outcomes.

	Motor Learning	Adjusted R^2^	P-value
**Δ MEP_max_**	Step Length Asymmetry	**0.418**	**0.014** *
	Step Time Asymmetry	**0.496**	**0.006** **
	Double Support Time Asymmetry	−0.035	0.446
	Net Metabolic Power	**0.666**	**0.001** **
**Δ Area Under the Curve**	Step Length Asymmetry	0.256	0.053
	Step Time Asymmetry	0.189	0.088
	Double Support Time Asymmetry	−0.067	0.591
	Net Metabolic Power	**0.649**	**0.001** **
**Δ Peak Slope**	Step Length Asymmetry	−0.007	0.358
	Step Time Asymmetry	0.233	0.064
	Double Support Time Asymmetry	−0.046	0.488
	Net Metabolic Power	**0.313**	**0.034** *
**Δ Resting Motor Threshold**	Step Length Asymmetry	−0.022	0.403
	Step Time Asymmetry	0.056	0.228
	Double Support Time Asymmetry	−0.099	0.942
	Net Metabolic Power	0.204	0.079

We performed linear regression analyses and calculated adjusted R^2^ values to examine the correlation between transcranial magnetic stimulation indices of corticospinal excitability and motor learning outcomes. Significance levels are denoted as

*** p < 0.001

** p < 0.01, and

* p < 0.05.

**Table 2.** Summary of the linear regression analyses performed between indices of AIH-induced enhancements in corticospinal excitability and motor savings outcomes.

	Motor Savings	Adjusted R^2^	P-value
**Δ MEP_max_**	Step Length Asymmetry	**0.625**	**0.001** **
	Step Time Asymmetry	**0.538**	**0.004** **
	Double Support Time Asymmetry	−0.030	0.429
	Net Metabolic Power	**0.645**	**0.001** **
**Δ Area Under the Curve**	Step Length Asymmetry	**0.290**	**0.041** *
	Step Time Asymmetry	**0.307**	**0.036** *
	Double Support Time Asymmetry	−0.100	0.997
	Net Metabolic Power	**0.693**	**<0.001** ***
**Δ Peak Slope**	Step Length Asymmetry	0.090	0.179
	Step Time Asymmetry	0.119	0.146
	Double Support Time Asymmetry	−0.041	0.468
	Net Metabolic Power	**0.406**	**0.015** *
**Δ Resting Motor Threshold**	Step Length Asymmetry	0.019	0.296
	Step Time Asymmetry	0.031	0.272
	Double Support Time Asymmetry	0.060	0.221
	Net Metabolic Power	0.231	0.065

We conducted linear regression analyses and computed adjusted R^2^ values to assess the relationship between transcranial magnetic stimulation indices of corticospinal excitability and motor savings outcomes. Significance levels are denoted as

*** p < 0.001

** p < 0.01, and

* p < 0.05.

## Data Availability

The authors confirm that the data supporting the findings of this study are fully available and presented in the supporting information of the manuscript. Correspondence and requests for materials should be addressed to A.Q.T.
