# Supplementary material for: Intermittent Hypoxia-Induced Enhancements in Corticospinal Excitability Predict Gains in Motor Learning and Metabolic Efficiency
Source: Res Sq. 2024 Apr 24:rs.3.rs-4259378. Preprint. [Version 1] doi: 10.21203/rs.3.rs-4259378/v1 (PMC11092812; doi:10.21203/rs.3.rs-4259378/v1)
Supplement: Supplement 1 [file NIHPPrs4259378v1-supplement-1.pdf]

Supplementary information

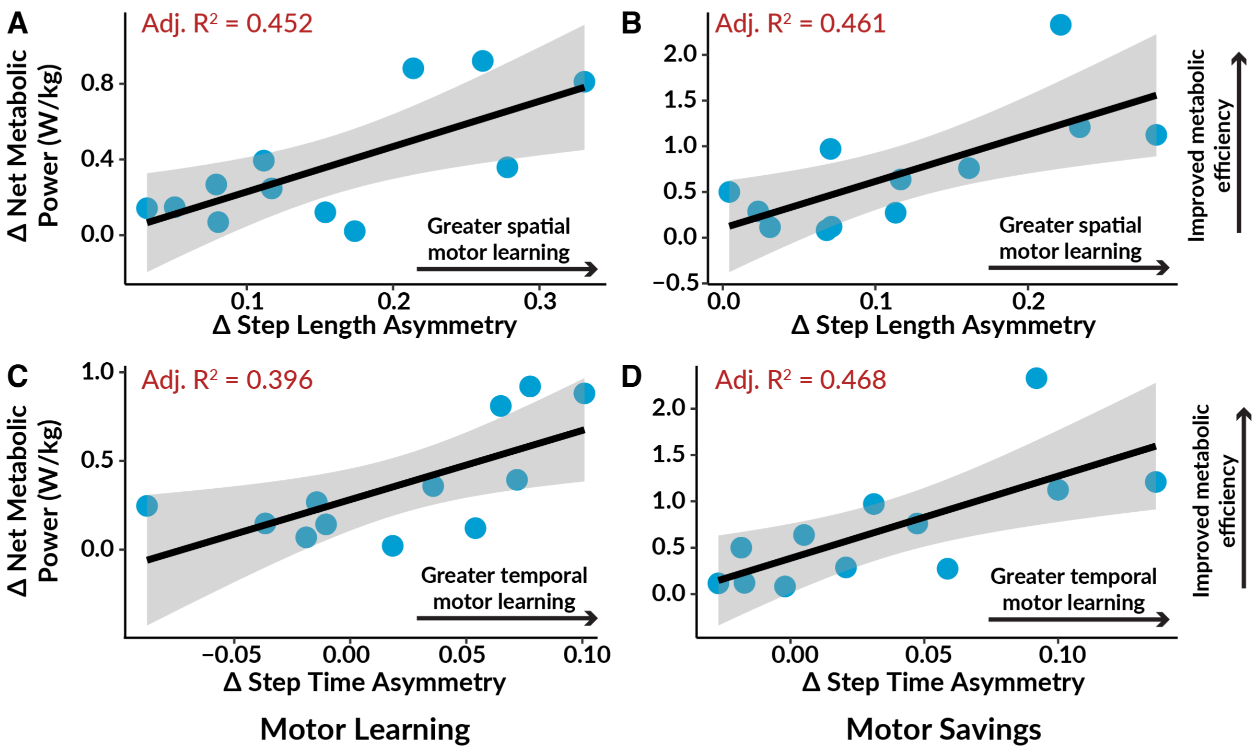

Supplementary Figure S1. Linear regressions among indices of spatiotemporal adaptation and their correlation with changes in net metabolic power. The red text indicates statistically significant adjusted R^2 values ($p < 0.05$). **A.** We observed a significant positive correlation between the decline in step length asymmetry during motor learning and the corresponding decrease in net metabolic power. **B.** The savings of reduced step length asymmetry demonstrated a significant correlation with the savings of lower net metabolic power. **C.** We observed a significant correlation between higher step time asymmetry during motor learning and decreased net metabolic power. **D.** The savings of step time asymmetry positively correlated with the savings of reduced net metabolic power. Individual data points are shown in blue, and the 95% confidence interval is represented by the grey-shaded area.

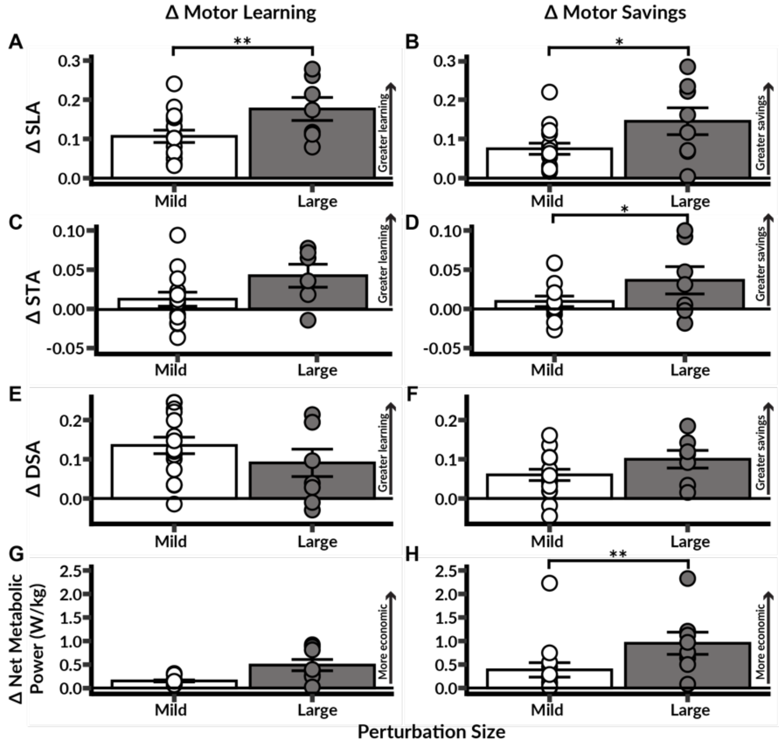

Supplementary Figure S2. Comparison of motor learning and motor savings between a mild and large perturbation group. We examined differences in motor learning (left) and motor savings (right) between a mild perturbation documented in Bogard et *al*., 2023 (1:1.5 belt speed ratio; white) and a large perturbation (1:2 belt speed ratio; grey). **A.** The large perturbation group displayed greater spatial motor learning than the mild perturbation group, as shown by larger reductions in step length asymmetry (SLA). **B.** The large perturbation group exhibited greater savings of lower SLA than the mild perturbation group. **C.** Both groups demonstrated an increase in step time asymmetry (STA) during motor learning. **D.** The large perturbation group showed greater savings of elevated STA compared to the mild perturbation group. **E.** Both groups adapted to reduce double support time asymmetry (DSA) during motor learning. **F.** Both groups demonstrated similar savings of lower DSA. **G.** Mean net metabolic power decreased during motor learning for both groups. **H.** The large perturbation group had greater reductions in net metabolic power from Adapt 1 to Adapt 2 compared to the mild perturbation group. The circles represent individual data. The bars indicate standard error. Significance levels are denoted as *** $p < 0.001$, ** $p < 0.01$, and * $p < 0.05$.

	Linear Mixed Model ANOVA			Tukey's *Post Hoc* Analyses	
	**Perturbation Size**	**Adaptation Period**	**Perturbation Size x Adaptation Period**	**Δ Motor Learning**	**Δ Motor Savings**
**Δ Step Length Asymmetry**	F(1, 21) = 6.70<br>**p = 0.017***	F(1, 21) = 37.28<br>**p < 0.001*****	F(1, 21) = 1.98<br>p = 0.174	t(23.01) = -2.83<br>**p = 0.010***	t(23.01) = -2.23<br>**p = 0.036***
**Δ Step Time Asymmetry**	F(1, 21) = 3.63<br>p = 0.071	F(1, 21) = 1.78<br>p = 0.196	F(1, 21) = 2.54<br>p = 0.126	t(24.46) = -1.39<br>p = 0.178	t(24.46) = -2.27<br>**p = 0.032***
**Δ Double Support Time Asymmetry**	F(1, 21) = 0.15<br>p = 0.705	F(1, 21) = 9.64<br>**p = 0.005****	F(1, 21) = 5.70<br>**p = 0.026***	t(29.05) = -1.32<br>p = 0.196	t(29.05) = 0.62<br>p = 0.538
**Δ Net Metabolic Power (W/kg)**	F(1, 20) = 7.51<br>**p = 0.013***	F(1, 20) = 9.95<br>**p = 0.005****	F(1, 20) = 0.95<br>p = 0.342	t(32.08) = -1.73<br>p = 0.093	t(32.08) = -2.82<br>**p = 0.008****

Supplementary Table S3. Retrospective comparisons between a mild vs. large perturbation size. Linear mixed model ANOVAs were used to assess a main effect of perturbation size (i.e., mild vs. large) and adaptation period (i.e., motor learning vs. motor savings), as well as an interaction between perturbation size and adaptation period (perturbation size x adaptation period). Tukey's post hoc analyses were used to assess significant perturbation size effects. Significance levels are denoted as *** $p < 0.001$, ** $p < 0.01$, and * $p < 0.05$.

Participant	Age (years)	Sex	Weight (kg)	Height (cm)
1	24	M	76.82	177.80
2	27	M	86.18	170.18
3	26	M	99.79	180.34
4	24	M	73.00	180.00
5	24	F	63.00	177.00
6	23	F	76.36	165.10
7	22	F	56.82	162.56
8	22	M	103.42	193.04
9	24	F	54.88	162.56
10	20	F	52.16	162.56
11	22	M	82.10	185.42
12	24	F	64.86	167.64
13	22	F	54.43	167.64

Supplementary Table S4. Demographic characteristics of the study participants. Thirteen able-bodied participants received AIH for 5 consecutive days at the University of Colorado, Boulder. All participants provided informed consent and had no prior history of cardiovascular disease, pulmonary complications, pain, syncope, or altitude sensitivity. All participants were recreationally active and were not undergoing physical therapy at the time of the study.
